# Intraoperative mild hyperoxia may be associated with improved survival after off-pump coronary artery bypass grafting: a retrospective observational study

**DOI:** 10.1186/s13741-022-00259-y

**Published:** 2022-07-19

**Authors:** Jae-Woo Ju, Hyun Woo Choe, Jinyoung Bae, Seohee Lee, Youn Joung Cho, Karam Nam, Yunseok Jeon

**Affiliations:** Department of Anesthesiology and Pain Medicine, Seoul National University Hospital, Seoul National University College of Medicine, Seoul, 03080 Republic of Korea

**Keywords:** Cardiac surgery, Coronary artery bypass grafting, Mortality, Outcome, Oxygen

## Abstract

**Background:**

The effect of hyperoxia due to supplemental oxygen administration on postoperative outcomes in patients undergoing cardiac surgery remains unclear. This retrospective study aimed to evaluate the relationship between intraoperative oxygen tension and mortality after off-pump coronary artery bypass grafting (OPCAB).

**Methods:**

The study included adult patients who underwent isolated OPCAB between July 2010 and June 2020. Patients were categorised into three groups based on their intraoperative time-weighted average arterial oxygen partial pressure (PaO_2_): normoxia/near-normoxia (< 150 mmHg), mild hyperoxia (150–250 mmHg), and severe hyperoxia (> 250 mmHg). The risk of in-hospital mortality was compared using weighted logistic regression analysis. Restricted cubic spline analysis was performed to analyse intraoperative PaO_2_ as a continuous variable. The risk of cumulative all-cause mortality was compared using Cox regression analysis.

**Results:**

The normoxia/near-normoxia, mild hyperoxia, and severe hyperoxia groups included 229, 991, and 173 patients (*n* = 1393), respectively. The mild hyperoxia group had a significantly lower risk of in-hospital mortality than the normoxia/near-normoxia (odds ratio [OR], 0.12; 95% confidence interval [CI], 0.06–0.22) and severe hyperoxia groups (*OR*, 0.06; 95% *CI*, 0.03–0.14). Intraoperative PaO_2_ exhibited a U-shaped relationship with in-hospital mortality in the non-hypoxic range. The risk of cumulative all-cause mortality was significantly lower in the mild hyperoxia group (hazard ratio, 0.72; 95% *CI*, 0.52–0.99) than in the normoxia/near-normoxia group.

**Conclusions:**

Maintaining intraoperative PaO_2_ at 150–250 mmHg was associated with a lower risk of mortality after OPCAB than PaO_2_ at < 150 mmHg and at > 250 mmHg. Future randomised trials are required to confirm if mildly increasing arterial oxygen tension during OPCAB to 150–250 mmHg improves postoperative outcomes.

## Background

During cardiac surgery, supplemental oxygen is conventionally employed with a high fraction of inspired oxygen (FiO_2_) to secure oxygen reserves and prevent perioperative hypoxia. The resultant supra-physiologic level of arterial oxygen partial pressure (PaO_2_) increases the oxygen gradient between capillaries and peripheral tissue, which may offset the reduced oxygen delivery (DO_2_) caused by hypothermia, fluid shift, myocardial dysfunction, blood loss, and anaemia during cardiac surgery (Spoelstra-de Man et al. 2015).

Previously held beliefs regarding the beneficial effects of supra-physiological oxygen tension have recently been questioned. Hyperoxia may increase oxidative stress by boosting the production of reactive oxygen species, thereby aggravating ischemia-reperfusion injury (Smit et al. 2016) and inducing vasoconstriction, both of which may decrease cardiac output (CO) and thus reduce DO_2_ (Bak et al. 2007). However, only a few studies have investigated this topic in patients undergoing cardiac surgery (Heinrichs et al. 2018). Such studies have exhibited heterogeneous designs, and most failed to demonstrate a difference in outcomes between normoxia and hyperoxia. Currently, there are no available guidelines for adequate oxygen therapy in patients undergoing cardiac surgery.

Meanwhile, frequent and sustained displacement and restraint of the heart during off-pump coronary artery bypass grafting (OPCAB) may further necessitate adequate oxygen therapy. However, there is a paucity of evidence regarding this setting (Heinrichs et al. 2018). We hypothesised that a mild supra-physiologic level of oxygen tension (i.e., mild hyperoxia) would improve postoperative mortality in patients undergoing OPCAB. The present study aimed to evaluate the relationship between intraoperative PaO_2_ and mortality following OPCAB.

## Methods

### Study design and population

This single-centre retrospective observational study involved patients who underwent isolated OPCAB at a tertiary university hospital between July 1, 2010, and June 20, 2020. The study protocol was approved by the Institutional Review Board of Seoul National University Hospital (approval no. 2007-010-1137) on July 7, 2020, and the requirement for written informed consent was waived due to the retrospective nature of the study. The study was conducted in accordance with the Strengthening the Reporting of Observational Studies in Epidemiology (STROBE) guidelines (von Elm et al. 2007).

Adult patients (≥ 18 years old) who underwent isolated OPCAB were included without a priori sample size calculation. The exclusion criteria were as follows: mechanical ventilation prior to surgery, fewer than four arterial blood gas measurements during surgery, repeat OPCABs in the same patient during the study period, and intraoperative extracorporeal membrane oxygenation.

### Anaesthetic management and intraoperative mechanical ventilation

Anaesthetic management was performed in accordance with the institutional protocol. Midazolam (0.1–0.2 mg/kg) and sufentanil (1.0–2.5 μg/kg) were administered to induce general anaesthesia. Rocuronium (0.6–1.2 mg/kg), vecuronium (0.1–0.2 mg/kg), or cisatracurium (0.1–0.2 mg/kg) was administered to facilitate tracheal intubation. A target-controlled infusion of propofol and remifentanil was utilised to maintain anaesthesia. The depth of anaesthesia was adjusted to maintain a bispectral index of 40–60. After tracheal intubation, patients received mechanical ventilation with an FiO_2_ of 0.4–0.5 and a tidal volume of 6–8 mL/kg. The respiratory rate was adjusted to maintain an end-tidal carbon dioxide partial pressure of 30–40 mmHg. If arterial oxygen saturation (SaO_2_) decreased to < 94% or PaO_2_ to < 80 mmHg, rescue therapy was performed in the following order: (1) alveolar recruitment manoeuvre, (2) applying a positive end-expiratory pressure of 5–10 cmH_2_O, and (3) increasing FiO_2_. There was no upper limit for the PaO_2_ target. Arterial blood gas analysis was performed using a point-of-care blood gas analyser (Gem Premier 3000; Instrumentation Laboratory, Bedford, MA, USA), and the measurements were carried out approximately every hour after the induction of anaesthesia and again after the above-mentioned rescue therapy. Pulmonary artery pressure, CO, and mixed venous oxygen saturation (SvO_2_) were continuously monitored using a pulmonary artery catheter (Swan-Ganz CCOmbo V 774HF75; Edwards Lifesciences, Irvine, CA, USA) connected to a monitoring device (Vigilance II™; Edwards Lifesciences). All patients were transferred to the intensive care unit (ICU) without extubation at the end of surgery. Mechanical ventilation was continued in the ICU, with an initial FiO_2_ of 0.6–0.8. Patients were extubated when SaO_2_ was maintained at > 94% and PaO_2_ at > 80 mmHg when FiO_2_ was < 0.5 and when positive end-expiratory pressure was < 8 cmH_2_O. The attending intensivist made the final decision regarding whether to wean the patient from mechanical ventilation.

### Study outcomes, study groups, and statistical analysis

The primary outcome was the risk of in-hospital mortality after OPCAB according to the intraoperative time-weighted average PaO_2_. Secondary outcomes included intraoperative haemodynamic and blood gas analysis data, cause of in-hospital death, acute kidney injury occurred within 7 days after surgery (defined based on the serum creatinine criteria of the Kidney Disease: Improving Global Outcomes definition) (Khwaja 2012), newly initiated renal replacement therapy after surgery, prolonged intubation (defined as cases where tracheal intubation was still required after postoperative 48 h), and the duration of supplemental oxygen after extubation, and risk of cumulative all-cause mortality according to the intraoperative time-weighted average PaO_2_.

**Fig. 1:**
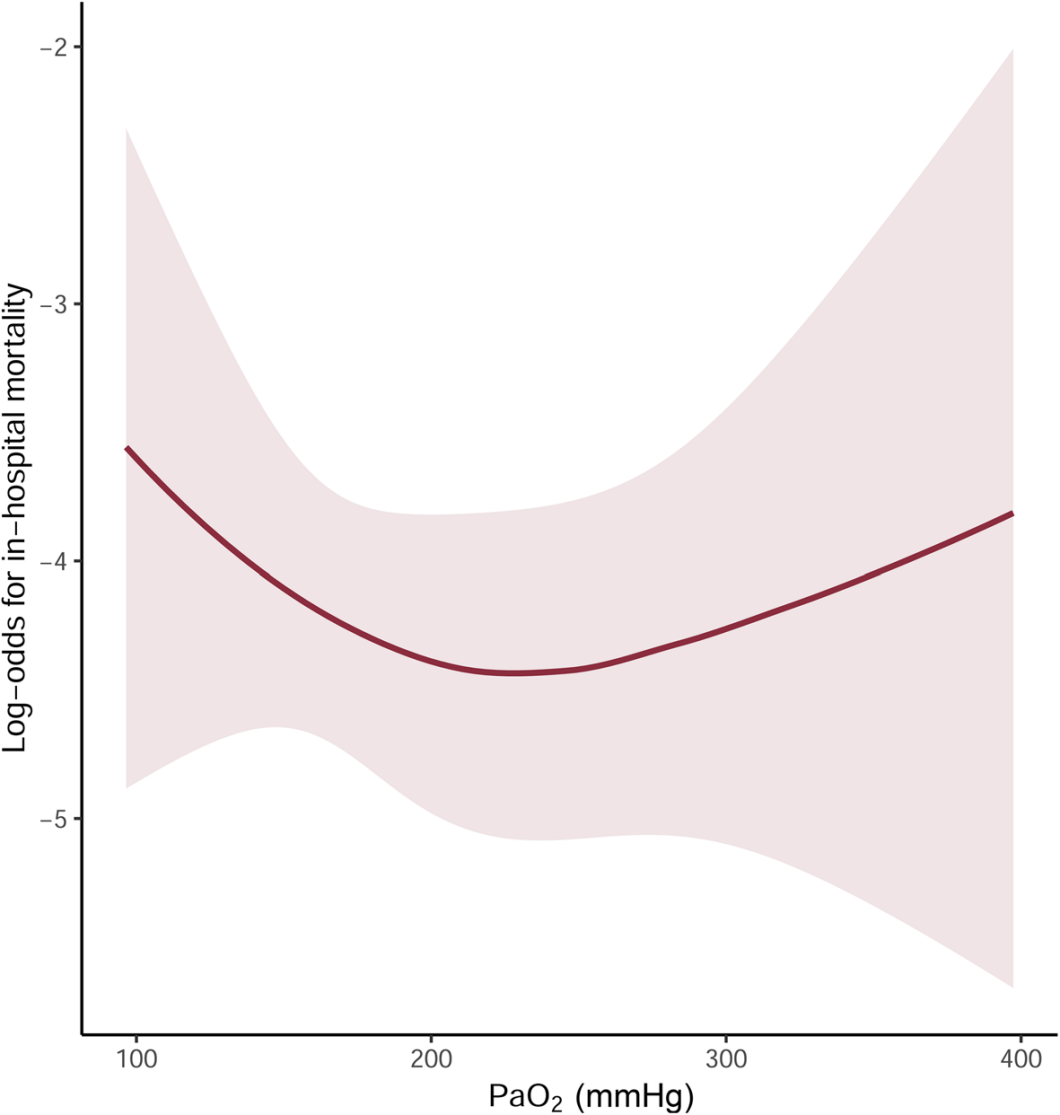
Preliminary, unadjusted restricted cubic spline model for log-odds of in-hospital mortality according to intraoperative time-weighted average PaO_2_. Bands indicate 95% confidence intervals. PaO_2_, arterial oxygen partial pressure

Intraoperative time-weighted average PaO_2_ was calculated as the area under the curve divided by the time interval between the first and last measurements. Before the analysis, patients were divided into three groups based on their time-weighted average PaO_2_: normoxia/near-normoxia (< 150 mmHg), mild hyperoxia (150–250 mmHg), and severe hyperoxia (> 250 mmHg). These cut-off values were determined based on a preliminary analysis using restricted cubic splines. The spline regression curve suggested a non-linear, “U-shaped” association between intraoperative time-weighted average PaO_2_ and postoperative in-hospital mortality in which an inflexion point was located around 200 mmHg (Fig. 1).

Considering a low event rate, the risk of in-hospital mortality was compared between the study groups using weighted logistic regression (Maalouf et al. 2018). The weights of the cases were calculated as follows:

$${w}_i=\frac{n}{k{n}_i},$$

where *w*_i_ represents the weight for class *i*, *n* represents the number of events in total, *k* represents the number of classes, and *n*_i_ represents the number of events in class *i* (King and Zeng 2001; Maalouf and Siddiqi 2014). After univariable logistic regression analysis, two multivariable analyses were performed. Model 1 was adjusted for variables included in the European System for Cardiac Operative Risk Evaluation (EuroSCORE) II model (Nashef et al. 2012): age, sex, renal impairment (categorized based on creatinine clearance calculated using the CockcroftGault formula: > 85 mL/min, 50–85 mL/min, < 50 mL/min, and preoperative dialysis regardless of creatinine clearance), extracardiac arteriopathy, previous cardiac surgery, chronic lung disease, diabetes mellitus on insulin, left ventricular ejection fraction (≤ 20%, 21–30%, 31–50%, and > 50%), recent myocardiac infarction (within 90 days before surgery), and pulmonary hypertention (defined as the first intraoperative pulmonary artery systolic pressure of > 30 mmHg measured using a pulmonary artery catheter). Poor mobility, critical preoperative state, New York Heart Association functional classification, and angina at rest were not included because complete and reliable data could not be obtained retrospectively by reviewing electronic medical records. Also, the model was not adjusted for active endocarditis because none of the patients had the condition at the time of surgery. In addition to the covariates used in model 1, model 2 was adjusted for patient characteristics (body mass index, smoking history), past medical history (hypertension, dyslipidaemia, atrial fibrillation, congestive heart failure, cerebrovascular disease), preoperative haematocrit, OPCAB-related factors (left main coronary artery disease, number of coronary artery anastomoses), duration of surgery, and year of surgery.

In addition, two multivariable restricted cubic spline regression analyses were used to analyse the non-linear continuous association between intraoperative time-weighted average PaO_2_ and in-hospital mortality (Gauthier et al. 2020). The multivariable spline models were adjusted for the same covariates included in models 1 and 2. Three knots were set at the 5th, 50th, and 95th percentiles of the time-weighted average PaO_2_ (Gauthier et al. 2020).

Intraoperative haemodynamic and blood gas analysis data (haematocrit, CO, cardiac index, and SvO_2_) were compared among the study groups using the Kruskal–Wallis test. These data were also analysed as time-weighted average values. Bonferroni correction was applied for pairwise comparisons when necessary (i.e. a statistical significance was examined at a *P*-value of < 0.05/3). The primary cause of in-hospital deaths was also investigated by reviewing the attending physician’s notes and death certificates from electronic medical records. Postoperative acute kidney injury, newly initiated renal replacement therapy, prolonged intubation, and the duration of supplemental oxygen after extubation were analyzed using a chi-squared test or the Kruskal–Wallis test where appropriate. For pairwise comparisons, Bonferroni correction was used (i.e., a statistical significance was evaluated at a *P*-value of < 0.05/3).

The risk of cumulative all-cause mortality following OPCAB according to study group was compared using Kaplan–Meier analysis, log-rank tests, and univariable and multivariable Cox regression analyses. The same multivariable procedure used for the logistic regression analyses was applied to construct two multivariable Cox regression models (models 1 and 2). Cox regression analyses were not adjusted for year of surgery.

All data were collected from electronic medical records using the Seoul National University Hospital Patients Research Environment (SUPREME) system, except for all-cause mortality data, which were obtained from the National Population Registry database of Korea. R (ver. 4.0.0; R Development Core Team, Vienna, Austria) was used for all statistical analyses. Continuous data are presented as mean (SD) or median (interquartile range [IQR]) and were compared using the analysis of variance or the Kruskal–Wallis test where appropriate. Categorical data are expressed as numbers (%) and were compared using Pearson’s chi-squared test or Fisher’s exact test, where appropriate. Statistical significance was set at a two-sided *P*-value of < 0.05.

## Results

Among the 1503 patients who underwent OPCAB during the study period, patients with fewer than four PaO_2_ measurements during surgery (*n* = 60), those who were mechanically ventilated prior to surgery (*n* = 12), those who underwent repeat OPCAB during the study period (*n* = 28), and those who received extracorporeal membrane oxygenation intraoperatively (*n* = 10) were excluded. Thus, data were analysed for 1393 patients. Based on the intraoperative time-weighted average PaO_2_, 229 (16.4%), 991 (71.1%), and 173 (12.4%) patients were classified into the normoxia/near-normoxia, mild hyperoxia, and severe hyperoxia groups, respectively. The median (IQR) intraoperative time-weighted average PaO_2_ values were 132 (121–141), 194 (175–214), and 292 (262–358) mmHg in the normoxia/near-normoxia, mild hyperoxia, and severe hyperoxia groups, respectively. Overall, the median (IQR) number of arterial blood gas measurements was 6 (5–7). The lowest intraoperative time-weighted PaO_2_ was 79 mmHg.

Baseline characteristics and perioperative data are shown in Table 1. Patients in the normoxia/near-normoxia group were older than those in the mild and severe hyperoxia groups. Hypertension, recent myocardial infarction, pulmonary hypertension, and emergency surgery were more frequent in the normoxia/near-normoxia group than in the mild and severe hyperoxia groups. The duration of surgery was also longer in the normoxia/near-normoxic group than in the other two groups.

**Table 1:** Baseline characteristics and perioperative data

	Normoxia/near-normoxia (*n* = 229)	Mild hyperoxia (*n* = 991)	Severe hyperoxia (*n* = 173)	*P*
*Demographic data*				
Age (y)	70 (63–75)	67 (60–73)	67 (58–73)	0.007
Female	61 (26.6%)	227 (22.9%)	35 (20.2%)	0.298
Body mass index (kg/m^2^)	25.4 (23.2–27.8)	24.0 (22.0–26.2)	23.1 (20.8–25.2)	< 0.001
Smoker	65 (28.4%)	311 (31.4%)	60 (34.7%)	0.401
*Medical history*				
Hypertension	164 (71.6%)	614 (62.0%)	91 (52.6%)	<0.001
Diabetes on insulin	33 (14.4%)	135 (13.6%)	21 (12.1%)	0.802
Dyslipidaemia	72 (31.4%)	267 (26.9%)	39 (22.5%)	0.135
Atrial fibrillation	16 (7.0%)	58 (5.9%)	10 (5.8%)	0.801
Recent myocardial infarction^a^	48 (21.0%)	148 (14.9%)	20 (11.6%)	0.023
Congestive heart failure	17 (7.4%)	56 (5.7%)	16 (9.2%)	0.159
Chronic lung disease	15 (6.6%)	38 (3.8%)	7 (4.0%)	0.186
Extracardiac arteriopathy	46 (20.1%)	181 (18.3%)	33 (19.1%)	0.807
Cerebrovascular disease	57 (24.9%)	225 (22.7%)	35 (20.2%)	0.543
Previous cardiac surgery	3 (1.3%)	24 (2.4%)	2 (1.2%)	0.376
*Preoperative clinical data*				
Left ventricular systolic function				0.536
Normal (EF > 50%)	155 (67.7%)	729 (73.6%)	122 (70.5%)	
Moderate (EF 31–50%)	54 (23.6%)	198 (20.0%)	38 (22.0%)	
Poor (EF 21–30%)	18 (7.9%)	52 (5.2%)	10 (5.8%)	
Very poor (EF ≤ 20%)	2 (0.9%)	12 (1.2%)	3 (1.7%)	
Pulmonary hypertension^b^	41 (17.9%)	113 (11.4%)	15 (8.7%)	0.008
Left main coronary artery disease	62 (27.1%)	254 (25.6%)	37 (21.4%)	0.399
Renal impairment^c^				0.444
Normal (CC > 85 mL/min)	57 (24.9%)	245 (24.7%)	38 (22.0%)	
Moderate (CC 50–85 mL/min)	108 (47.2%)	485 (48.9%)	82 (47.4%)	
Severe (CC < 50 mL/min)	47 (20.5%)	191 (19.3%)	32 (18.5%)	
On dialysis regardless of CC	17 (7.4%)	70 (7.1%)	21 (12.1%)	
Haematocrit (%)	38 (5)	38 (4)	37 (4)	0.186
*Surgery profile*				
Emergency	18 (7.9%)	38 (3.8%)	4 (2.3%)	0.010
No. of coronary artery anastomoses	3 (3–4)	4 (3–4)	3 (3–4)	0.368
Duration of surgery (min)	365 (317–400)	360 (325–395)	325 (295–370)	< 0.001

Values are expressed as mean (standard deviation), median (interquartile range), or number (%)

*EF* Ejection fraction, *CC* Creatinine clearance

^a^Myocardial infarction within 90 days prior to surgery

^b^Defined as the first intraoperative pulmonary artery systolic pressure measurement of > 30 mmHg

^c^Based on creatinine clearance calculated using Cockcroft-Gault formula

**Fig. 2:**
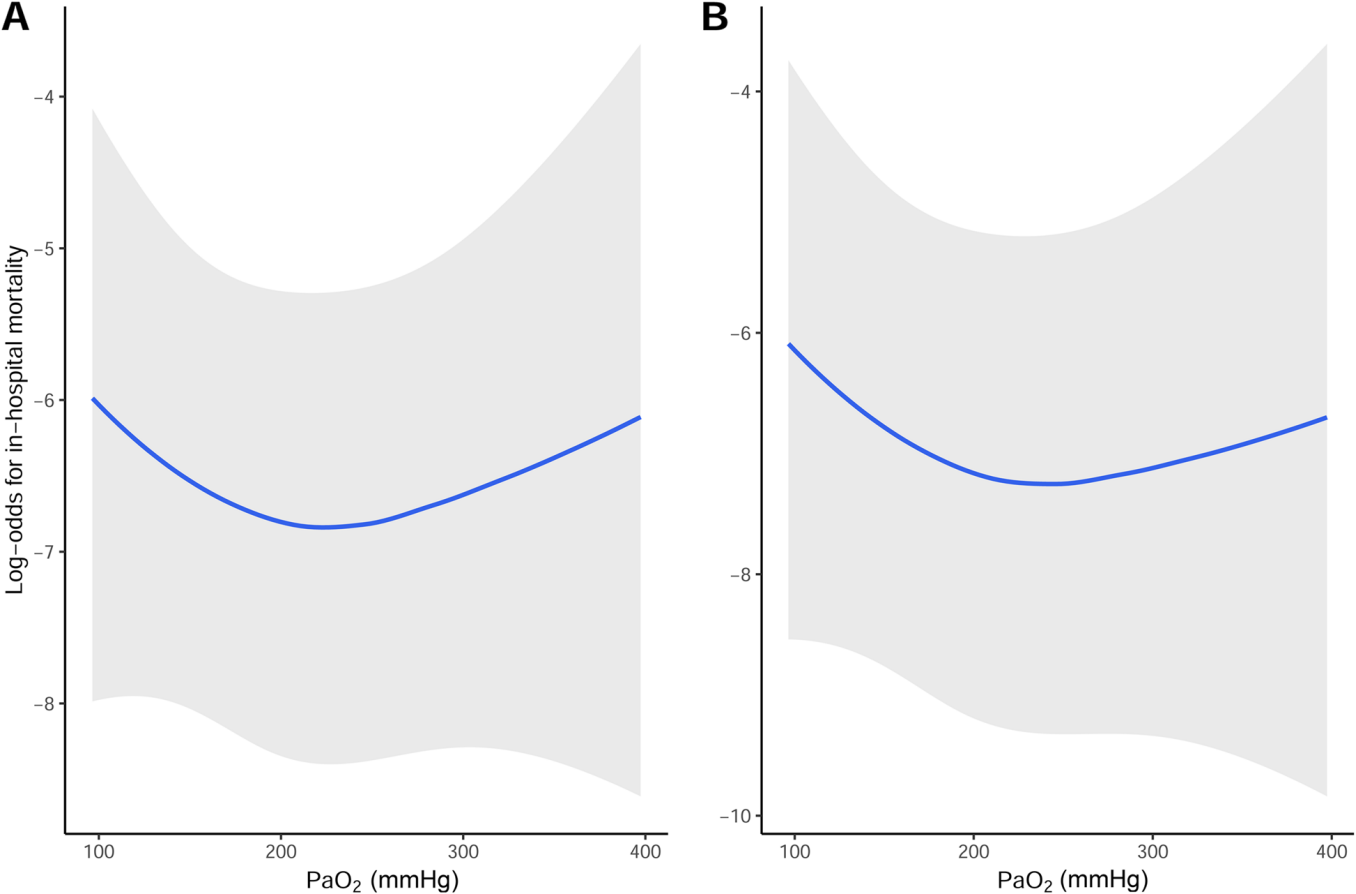
Multivariable restricted cubic spline models for log-odds of in-hospital mortality according to intraoperative time-weighted average PaO_2_. **A** Adjusted for the covariates in model 1. **B** Adjusted for the covariates in model 2. Bands indicate 95% confidence intervals. PaO_2_, arterial oxygen partial pressure

The overall in-hospital mortality rate after OPCAB was 1.4% (20/1393). In-hospital mortality rates were 2.6% (6/229), 1.0% (10/991), and 2.3% (4/173) in the normoxia/near-normoxia, mild hyperoxia, and severe hyperoxia groups, respectively. The results of the weighted logistic regression analysis are summarised in Table 2. Patients in the mild hyperoxia group were at a significantly lower risk of in-hospital mortality than those in the normoxia/near-normoxia group in all weighted logistic regression models (model 1: odds ratio [OR], 0.24; 95% confidence interval [CI], 0.16–0.28; *P* < 0.001; and model 2: *OR*, 0.12; 95% *CI*, 0.06–0.22; *P* <0.001). In addition, the risk of in-hospital mortality was significantly lower in the mild hyperoxia group than in the severe hyperoxia group (model 1: *OR*, 0.16; 95% *CI*, 0.10–0.26; *P* < 0.001; and model 2: *OR*, 0.06; 95% *CI*, 0.03–0.14; *P* < 0.001; data not shown in Table 2). Similar results were observed for the multivariable restricted cubic spline curves (Fig. 2). The spline curves revealed a non-linear, U-shaped relationship between intraoperative time-weighted average PaO_2_ and in-hospital mortality in which an inflexion point was located at approximately 200 mmHg.

**Table 2:** Weighted logistic regression model for in-hospital mortality after off-pump coronary artery bypass grafting

	Univariable	*P*	Multivariable 1^a^	*P*	Multivariable 2^b^	*P*
	OR (95% *CI*)		OR (95% *CI*)		OR (95% *CI*)	
Normoxia/near-normoxia	Reference		Reference		Reference	
Mild hyperoxia	0.38 (0.29–0.50)	< 0.001	0.24 (0.16–0.28)	< 0.001	0.12 (0.06–0.22)	< 0.001
Severe hyperoxia	0.88 (0.62–1.25)	0.477	1.54 (0.89–2.65)	0.120	1.91 (0.80–4.55)	0.147

*OR* Odds ratio, *CI* Confidence interval

^a^Model 1 was adjusted for age, sex, renal impairment, extracardiac arteriopathy, previous cardiac surgery, chronic lung disease, diabetes mellutus on insulin, left ventricular ejection fraction, recent myocardiac infarction (within 90 days), and pulmonary hypertention

^b^Model 2 was adjusted for all variables used in model 1 and body mass index, smoking history, hypertension, dyslipidaemia, atrial fibrillation, congestive heart failure, cerebrovascular disease, preoperative haematocrit, left main coronary artery disease, number of coronary artery anastomoses, duration of surgery, and year of surgery

The intraoperative haemodynamic and blood gas analysis results are presented in Table 3. Although the differences were statistically significant in the nonparametric Kruskal–Wallis test, intraoperative haematocrit, CO, and cardiac index were clinically similar between the groups (Table 3). SvO_2_ was significantly higher in the mild hyperoxia group (median [IQR], 70% [66–74]) than in the normoxia group (67% [63–71]; *P* < 0.001). The causes of in-hospital mortality are described in Table 4. The most common cause of death was infection.

**Table 3:** Comparison of intraoperative hemodynamic and blood gas analysis data among the study groups

	Normoxia/near-normoxia (*n* = 229)	Mild hyperoxia (*n* = 991)	Severe hyperoxia (*n* = 173)	*P*
PaO_2_ (mmHg)	132 (121–141)	194 (175–214)	292 (262–358)	< 0.001
Haematocrit (%)	31 (29–34)	30 (28–32)	30 (28–33)	0.027
Cardiac output (L/min)^a^	3.7 (3.1–4.2)	3.6 (3.1–4.1)	3.4 (3.0–3.8)	< 0.001
Cardiac index (L/min/m^2^)^a^	2.1 (1.9–2.4)	2.1 (1.9–2.4)	2.0 (1.9–2.2)	< 0.001
SvO_2_ (%)^b^	67 (63–71)	70 (66–74)	72 (69–76)	< 0.001

*PaO*_*2*_ Arterial oxygen partial pressure, *SvO*_*2*_ Mixed venous oxygen saturation

^a^Twenty-six missing values

^b^45 missing values

**Table 4:** Causes of in-hospital death according to the study groups

Cause of in-hospital death	*n*
*Normoxia/near-normoxia*	*6 died out of 229 (2.6%)*
Infection	2
Brain infarct	1
Fatal ventricular arrhythmia	1
Hypovolemic shock	1
Unknown or unspecified	1
*Mild hyperoxia*	*10 died out of 991 (1.0%)*
Infection	4
Brain infarct	1
Cardiogenic shock	1
Ischemic colitis	1
Iatrogenic	1
Unknown or unspecified	2
*Severe hyperoxia*	*4 died out of 173 (2.3%)*
Infection	2
Coronary vasospasm	1
Rhabdomyolysis	1

The results of other secondary postoperative outcomes are summarized in Table 5. There was no significant difference in the occurrence of acute kidney injury and newly initiated renal replacement therapy after surgery among the groups. The incidence of prolonged intubation and the duration of supplemental oxygen after extubation were significantly greater in the mild hyperoxia group compared to the normoxia/near-normoxia group (both pairwise *P* <0.001); they were not different significantly between the mild hyperoxia and severe hyperoxia groups (a pairwise *P* = 0.377 and 0.042, respectively; not shown in Table 5).

**Table 5:** Comparison of secondary outcomes after off-pump coronary artery bypass grafting between the study groups

	Normoxia/near-normoxia (*n* = 229)	Mild hyperoxia (*n* = 991)	Severe hyperoxia (*n* = 173)	*P*
Acute kidney injury^a^	67 (29.3%)	272 (27.4%)	34 (19.7%)	0.066
Newly initiated renal replacement therapy	7 (3.1%)	25 (2.5%)	4 (2.3%)	0.874
Prolonged intubation^b^	25 (11%)	43 (4.3%)	5 (2.9%)	< 0.001
Supplemental oxygen therapy after extubation (hour)	83 (51–120)	61 (35–94)	53 (33–80)	< 0.001

Values are expressed as median (interquartile range) or number (%)

^a^Defined based on the serum creatinine criteria of the kidney disease: improving Global Outcomes definition

^b^Defined as cases where tracheal intubation was still required after 48 postoperative hours

**Fig. 3:**
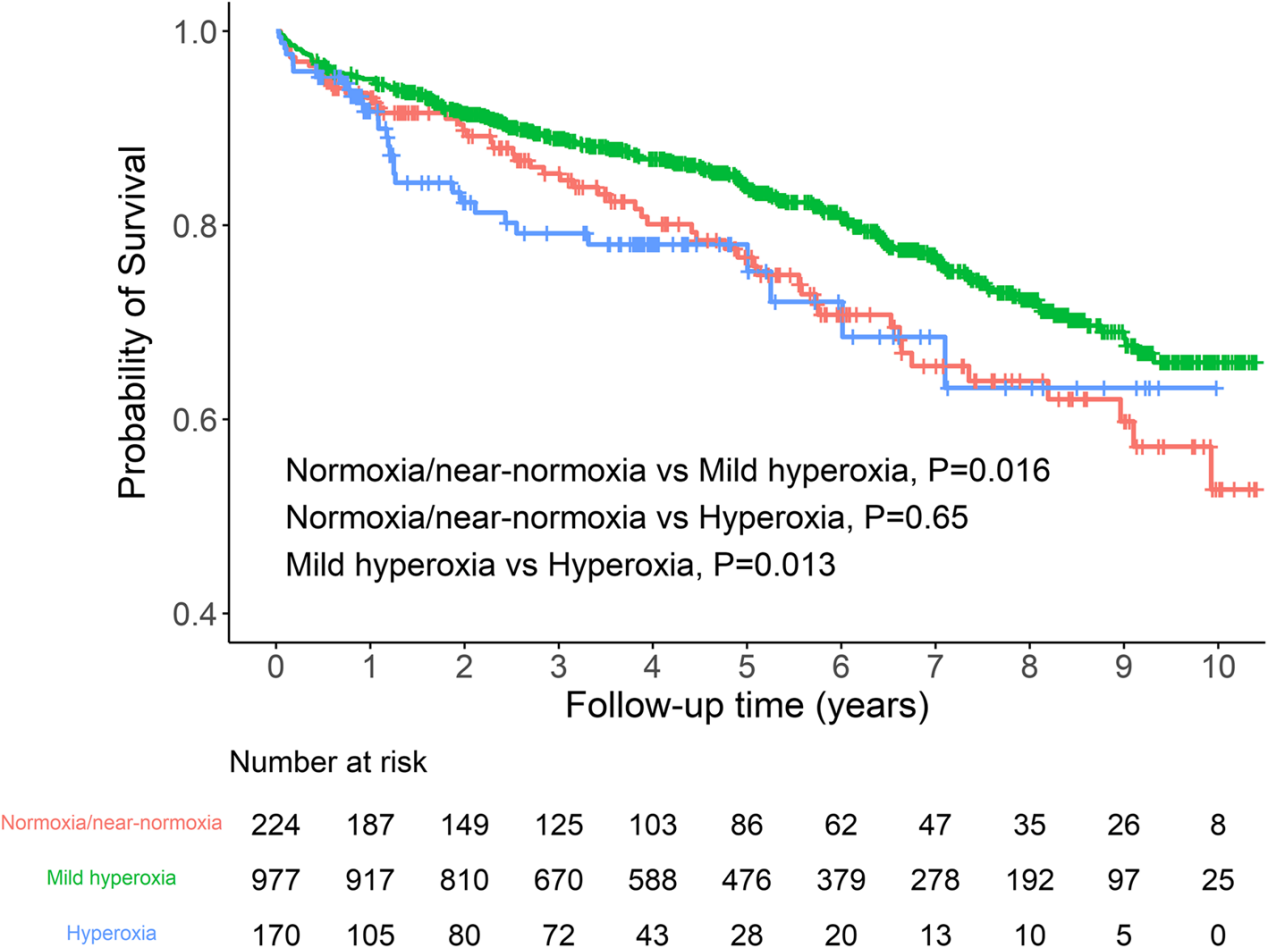
Kaplan–Meier curves for postoperative all-cause mortality according to study group. Tick marks indicate censoring

Kaplan–Meier curves for cumulative all-cause mortality are shown in Fig. 3. The survival data of 22 patients were not retrieved from the National Population Registry database. The median (IQR) duration of follow-up of the remaining patients was 4.3 (2.1–7.0) years. Postoperative cumulative all-cause mortality was significantly lower in the mild hyperoxia group than in the normoxia/near-normoxia (log-rank test, pairwise comparison; *P* = 0.016) and severe hyperoxia groups (*P* = 0.013). In multivariable Cox regression model 1, the risk of postoperative mortality was lower in the mild hyperoxia group than in the normoxia/near-normoxia group, but the difference was not statistically significant (hazard ratio, 0.82; 95% CI, 0.60–1.11; *P* = 0.199; Table 6). In model 2, the mild hyperoxia group exhibited a significantly lower risk of mortality than the normoxia/near-normoxia group (hazard ratio, 0.72; 95% *CI*, 0.52–0.99; *P* = 0.048). When compared with the severe hyperoxia group, the adjusted hazard ratios of the mild hyperoxia group were 0.58 (95% *CI*, 0.39–0.86; *P* = 0.007) in model 1 and 0.69 (95% CI, 0.46–1.03; *P* = 0.071) in model 2 (data not shown in Table 6).

**Table 6:** Cox regression models for cumulative all-cause mortality after off-pump coronary artery bypass grafting^a^

	Univariable	*P*	Multivariable 1^b^	*P*	Multivariable 2^c^	*P*
	HR (95% *CI*)		HR (95% *CI*)		HR (95% *CI*)	
Normoxia/near-normoxia	Reference		Reference		Reference	
Mild hyperoxia	0.69 (0.51–0.93)	0.016	0.82 (0.60–1.11)	0.199	0.72 (0.52–0.99)	0.048
Severe hyperoxia	1.12 (0.72–1.74)	0.627	1.41 (0.89–2.24)	0.141	1.05 (0.65–1.69)	0.851

*HR* Hazard ratio, *CI* Confidence interval

^a^Twenty-two patients were not included because their survival data were not available

^b^Model 1 was adjusted for variables consisting of the European System for Cardiac Operative Risk Evaluation II model (see the “Methods” section)

^c^Model 2 was adjusted for all variables used in model 1 and demographic data and perioperative variables listed in Table 1 (see the “Methods” section)

In this study, we investigated the relationship between intraoperative oxygen tension and mortality after OPCAB. A mildly hyperoxic level of intraoperative arterial oxygen tension was associated with improved outcomes after OPCAB when compared to normoxic, near-normoxic, and severely hyperoxic levels. Patients with intraoperative time-weighted average PaO_2_ levels between 150 mmHg and 250 mmHg had a significantly lower risk of in-hospital mortality than those with time-weighted average PaO_2_ levels 150 mmHg and 250 mmHg. Furthermore, intraoperative PaO_2_ exhibited a U-shaped relationship with in-hospital mortality in the non-hypoxic range.

Maintaining adequate DO_2_ is of utmost concern for patients undergoing cardiac surgery. Decreased perioperative DO_2_ is associated with complications after cardiac surgery, including neurologic injury (Hogue Jr. et al. 1999; Bahrainwala et al. 2011; Magruder et al. 2018; Murphy et al. 2009) and renal impairment (de Somer et al. 2011; Ranucci et al. 2005; Magruder et al. 2015). To optimise perioperative DO_2_, physicians tend to focus only on CO, haemoglobin (Hb) concentration, and SaO_2_. In contrast, PaO_2_ has been of less interest because its theoretical contribution to DO_2_ and arterial oxygen content (CaO_2_) is limited according to the following equation (Shepherd and Pearse 2009):

$${\displaystyle \begin{array}{c}{DO}_2= CO\times {CaO}_2\\ {}= CO\times \left(1.34\times Hb\times {SaO}_2+0.0034\times {PaO}_2\right).\end{array}}$$

In addition, most previous studies have emphasised the importance of CO and Hb concentrations rather than PaO_2_ (Hogue Jr. et al. 1999; Bahrainwala et al. 2011; Ranucci et al. 2005). In this study, we demonstrated that postoperative mortality may differ according to intraoperative PaO_2_ strata given similar Hb concentrations and CO. From our analysis of the causes of death, we could not identify any clues to the mechanism underlying this finding. Nonetheless, higher SvO_2_ (indicating a higher DO_2_) may explain in part the improved postoperative mortality observed in the mild hyperoxia group (see the “Results” section). Similar results were reported by Legrand et al. (2014). In their study, median central venous oxygen saturation increased from 71% to 84% after increasing FiO_2_ from 0.4 to 1.0 in critically ill patients (Legrand et al. 2014). The increase in central venous oxygen saturation was not fully explained by CO, Hb level, or SaO_2_; rather, it was considerably accounted for by PaO_2_ (Legrand et al. 2014). Likewise, Yu et al. (2006) observed a significant increase in tissue oxygen partial pressure after increasing FiO_2_ in critically ill patients. Taken together, these findings indicate that dissolved oxygen (or PaO_2_) may contribute to DO_2_ more than expected in real-world practice. According to the aforementioned equation, in a hypothetical patient with Hb concentration of 10 g/dL and an SaO_2_ of 100%, an isolated change of 0.5 g/dL in Hb concentration or 5% in SaO_2_ is equivalent to a PaO_2_ change of 197 mmHg. This calculation implies that a large increase in PaO_2_ is required to obtain a clinically meaningful increase in DO_2_. However, in our study, we observed that even a mild increase in intraoperative PaO_2_ may result in improved survival after OPCAB. Considering that transfusion may be associated with poor postoperative outcomes (Nam et al. 2020; Rohde et al. 2014; Vlaar et al. 2011) and that SaO_2_ remains 100% or nearly 100% during intraoperative mechanical ventilation, increasing FiO_2_ (thereby increasing PaO_2_) may be a simple and efficient alternative method for physicians to improve DO_2_ during cardiac surgery.

In our study, severe intraoperative hyperoxia (PaO_2_ > 250 mmHg) was associated with an increased risk of mortality compared to mild hyperoxia (PaO_2_ 150–250 mmHg). Moreover, on the spline curves, the risk of in-hospital mortality exhibited a U-shaped pattern. The risk declined as intraoperative PaO_2_ increased from the normoxic level to approximately 200 mmHg, following which it began to increase. Similar results were reported by Helmerhorst et al. (2017). In their multicentre observational cohort study of more than 14,000 ICU patients, various PaO_2_ metrics used to define hyperoxia during ICU admission exhibited a U-shaped relationship with in-hospital mortality. However, their PaO_2_ inflexion point appeared earlier, at approximately 150 mmHg. Given the absence of a clear definition of hyperoxia (Heinrichs et al. 2018), future studies seeking a hyperoxic threshold beyond which clinical outcomes worsen are warranted. Meanwhile, in a recent meta-analysis of eight randomised trials performed in post-cardiac arrest patients and patients with acute respiratory distress syndrome, trauma, septic shock, and major organ failure (Zhao et al. 2021), there was no difference in 30-day mortality between different PaO_2_ goals of < 90 mmHg, 90–150 mmHg, and > 150 mmHg. However, survival curves suggested that a PaO_2_ level of > 150 mmHg may be inferior to the other levels (Zhao et al. 2021). To directly compare these results with ours may not be adequate, because the study population and the timing of oxygen exposure are very different. Nonetheless, it is highly likely that there is an optimal PaO_2_ range associated with the best clinical outcomes in various clinical settings.

In this study, CO levels were comparable between the normoxia/near-normoxia and mild hyperoxia groups, whereas the CO level in the severe hyperoxia group (PaO_2_ > 250 mmHg) was significantly lower than that in the other groups (pairwise comparisons, not shown in the “Results” section). This may be important given that previous studies have reported that significant hyperoxia (PaO_2_ 450–550 mmHg) increases systemic vascular resistance, thus decreasing CO (Harten et al. 2005; Inoue et al. 2002). In another study, Smit et al. (2016) compared a PaO_2_ target of 200–220 mmHg during cardiopulmonary bypass and 130–150 mmHg during ICU admission (similar to the mild hyperoxia group in our study) to a lower target of 130–150 mmHg during cardiopulmonary bypass and 80–100 mmHg in the ICU (similar to the normoxia/near-normoxia group in our study). The resultant systemic vascular resistance and CO did not differ between the two targets. These results are concordant with our finding that mild hyperoxia (PaO_2_ of 150–250 mmHg) increased SvO_2_ without a decrease in CO. To date, the PaO_2_ threshold beyond which CO begins to decrease remains unknown.

Our results should be interpreted with caution for several reasons. First, this study was retrospective in nature, and the results may indicate merely an association, not a cause-effect relationship between intraoperative hyperoxia and mortality after OPCAB. Although we adjusted for a large set of clinical covariates to offset this drawback, potential confounders may still be in play. Indeed, we could not address some of the EuroSCORE II variables. Randomised controlled trials should therefore be conducted. An ongoing study aims to compare the length of hospital stay and various clinical outcomes after OPCAB between patients receiving two different levels of intraoperative FiO_2_ (ClinicalTrials.gov identifier, NCT03945565). Second, since FiO_2_ was usually set to 0.4–0.5 in this study, the difference in PaO_2_ may have stemmed from individual lung conditions, such as diffusion capacity or ventilation/perfusion ratio, which may have confounded our results. Third, we only compared SvO_2_ among the study groups and could not calculate DO_2_. Although DO_2_ is reflected as SvO_2_, it is accurate to say that SvO_2_ indicates a balance between oxygen supply and demand (Shepherd and Pearse 2009).

## Conclusions

In conclusion, intraoperative mild hyperoxia (PaO_2_ of 150–250 mmHg) was significantly associated with a significantly lower risk of in-hospital mortality after OPCAB than normoxia/near-normoxia (PaO_2_ < 150 mmHg) and severe hyperoxia (PaO_2_ > 250 mmHg). Intraoperative PaO_2_ exhibited a U-shaped relationship with postoperative mortality in the non-hypoxic range. Thus, randomized trials are required to confirm if maintaining a mildly supra-physiologic level of arterial oxygen tension improves postoperative outcomes in patients undergoing OPCAB.

## Data Availability

The datasets used and/or analysed during the current study are available from the corresponding author on reasonable request.

## Abbreviations

CaO_2_: Arterial oxygen content
CI: Confidence interval
EuroSCORE: European System for Cardiac Operative Risk Evaluation
FiO_2_: Fraction of inspired oxygen
Hb: Haemoglobin
ICU: Intensive care unit
IQR: Interquartile
OR: Odds ratio
PaO_2_: Arterial oxygen partial pressure
SaO_2_: Arterial oxygen saturation
STROBE: Strengthening the Reporting of Observational Studies in Epidemiology
SUPREME: Seoul National University Hospital Patients Research Environment
SvO_2_: Mixed venous oxygen saturation
